# Inflammasomes in cancer: a double-edged sword

**DOI:** 10.1007/s13238-013-0001-4

**Published:** 2014-01-29

**Authors:** Ryan Kolb, Guang-Hui Liu, Ann M. Janowski, Fayyaz S. Sutterwala, Weizhou Zhang

**Affiliations:** 1 Department of Pathology, Carver College of Medicine, University of Iowa, Iowa City, IA 52242 USA; 2 National Laboratory of Biomacromolecules, Institute of Biophysics, Chinese Academy of Sciences, Beijing, 100101 China; 3 Gene Expression Laboratory, Salk Institute for Biological Studies, 10010 North Torrey Pines Road, La Jolla, CA 92037 USA; 4 Graduate Program in Immunology, Carver College of Medicine, University of Iowa, Iowa City, IA 52242 USA; 5 Department of Internal Medicine, Carver College of Medicine, University of Iowa, Iowa City, IA 52242 USA; 6 Veterans Affairs Medical Center, Iowa City, IA 52241 USA

**Keywords:** inflammasome, cancer, inflammation

## Abstract

Chronic inflammatory responses have long been observed to be associated with various types of cancer and play decisive roles at different stages of cancer development. Inflammasomes, which are potent inducers of interleukin (IL)-1β and IL-18 during inflammation, are large protein complexes typically consisting of a Nod-like receptor (NLR), the adapter protein ASC, and Caspase-1. During malignant transformation or cancer therapy, the inflammasomes are postulated to become activated in response to danger signals arising from the tumors or from therapy-induced damage to the tumor or healthy tissue. The activation of inflammasomes plays diverse and sometimes contrasting roles in cancer promotion and therapy depending on the specific context. Here we summarize the role of different inflammasome complexes in cancer progression and therapy. Inflammasome components and pathways may provide novel targets to treat certain types of cancer; however, using such agents should be cautiously evaluated due to the complex roles that inflammasomes and pro-inflammatory cytokines play in immunity.

## Introduction

Inflammasomes are multimolecular complexes that promote inflammation and inflammatory cell death (referred to as pyroptosis) through the activation of cysteine protease caspase-1 in response to both microbial insults to the host and endogenous damage-associated molecular patterns (DAMPS) such as uric acid and extracellular ATP. Inflammasome complexes are typically comprised of an NLR which interacts directly with caspase-1 through a caspase activation and recruitment domain (CARD) or via an adapter protein, usually apoptosis-associated speck-like protein containing a caspase recruitment domain (ASC; also known as Pycard), which links the NLR to caspase-1. Different inflammasomes are named for the NLR present in the complex, such as the NLRP3-, NLRC4-, NLRP1-, or NLRP6-inflammasome. In addition to the NLR inflammasomes, absent in melanoma 2 (AIM2), one of the PYHIN family members, is the main component of the AIM2 inflammasome (Schroder and Tschopp, 2010; Di Virgilio, 2013).

**Figure 1:**
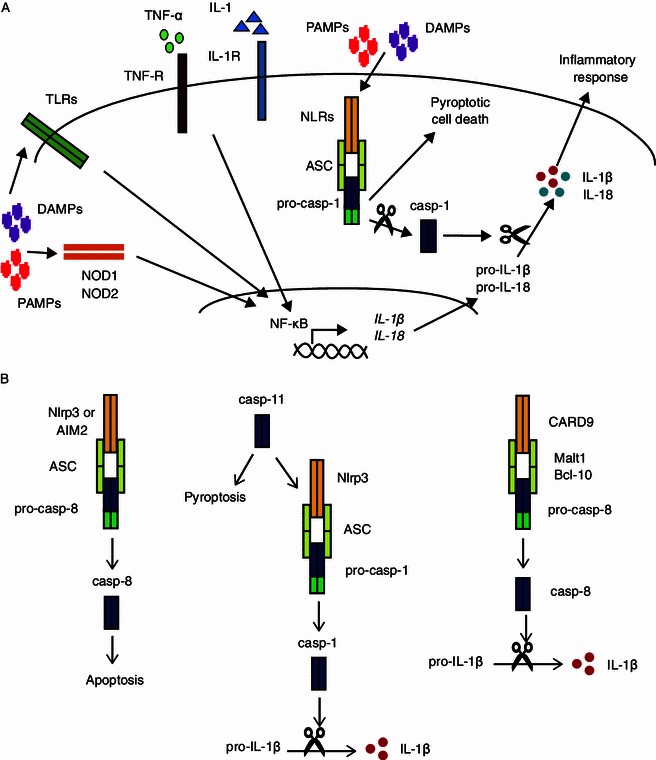
Inflammasome activation and signaling. (A) Inflammasomes are activated in two steps. First, priming induces the expression of pro-IL-1β and pro-IL-18 through the activation of NF-κB. NF-κB is activated by TNF-α and IL-1 or through sensing of “danger signals” (PAMPs and DAMPs) by TLRs or NOD1/2. Priming also readies the inflammasomes for activation through other unknown mechanism. The second step involves the sensing of PAMPs and DAMPs by NLRs (NLRP3, NLRC4, etc.) or AIM2 through mechanisms that are not fully understood. Some NLRs, such as NLRP3 and NLRC4, interact with pro-caspase-1 through ASC, while others, such as NLRP1 can interact directly with caspase-1. Activation of NLRs results in the activation of pro-caspase-1, which when cleaved can catalyze the proteolytic cleavage and activation of IL-1β and IL-18. Activation of caspase-1 can also induce pyroptotic cell death, though cleavage of caspase-1 is not required for this process. (B) Non-canonical inflammasomes involve activation of caspase-8 and caspase-11 which can lead to induction of pyroptosis, apoptosis and activation of IL-1β

Canonical inflammasome activation is a two-step process (Fig. 1A). The first step involves the induction of mRNA and protein expression of pro-IL-1β and pro-IL-18, a procedure mediated by NF-κB activation. NF-κB-mediated transcription of pro-IL-1β and pro-IL-18 is induced by activation of toll-like receptors (TLRs), NOD1 or NOD2 by DAMPS or pathogen- associated molecular patterns (PAMPS) or signaling by tumor necrosis factor-α (TNF-α) or IL-1 through their respective receptors (Chen and Nunez, 2011). Priming also readies inflammasomes for activation; one possible mechanism for this is postulated to be the upregulation of NLRP3 expression in response to LPS. However, a study by Shcroder et al. showed that LPS can augment inflammasome activation independently from NLRP3 expression, suggesting that other mechanism for priming exists (Bauernfeind et al., 2009; Schroder et al., 2012). Indeed, a recent study by Juliana et al. showed that mitochondria-derived reactive oxygen species (ROS) can prime the NLRP3 inflammasome for activation by de-ubiquitination of NLRP3 through a mechanism involving TLR4/MyD88 signaling. This study also indicated that signaling by ATP can prime the NLRP3 inflammasome through de-ubiquitination (Juliana et al., 2012). The second step in inflammasome activation comes from NLRs sensing PAMPs or DAMPs. Different inflammasomes are activated by different signals. For example, the NLRP3 inflammasome can be activated by various signals including bacterial pore forming toxins, extracellular ATP, uric acid crystals, and asbestos (Mariathasan et al., 2006; Martinon et al., 2006; Dostert et al., 2008; Lamkanfi and Dixit, 2009; Bauernfeind et al., 2011; Franchi and Nunez, 2012). The NLRC4 inflammasome is activated in response to cytosolic flagellin from various gram-negative bacteria or components of the bacterial type three or four secretion systems (Mariathasan et al., 2004; Franchi et al., 2006; Miao et al., 2006; Zamboni et al., 2006; Suzuki et al., 2007; Miao et al., 2008; Chen and Nunez, 2011). Cytosolic double-stranded DNA from pathogens such as *Franscisella tularensis* and *Listeria monocytogenes* has been demonstrated to activate the AIM2 inflammasome (Fernandes-Alnemri et al., 2009; Hornung et al., 2009; Fernandes-Alnemri et al., 2010; Kim et al., 2010; Rathinam et al., 2010). For a comprehensive review of inflammasome activators, please refer to the recent article (Bauernfeind and Hornung, 2013). However, the mechanism of how different inflammasomes are activated by these various signals is not fully understood. Sensing of danger signals by NLRs or AIM2 leads to the oligomerization and activation of caspase-1. Caspase-1 activates pro-IL-1β or pro-IL-18 by proteolytic cleavage, which in turn promotes inflammation and regulates immune responses (Zitvogel et al., 2012; Di Virgilio, 2013). Caspase-1 activation can also induce pyroptosis, an inflammatory cell death that is accompanied by the release of IL-1β and IL-18 which elicits local inflammation (Miao et al., 2011). Recent evidence has shown that non-canonical activation of inflammasomes involves caspase-8 or caspase-11. Kayagaki et al. showed that the activation of caspase-11 in infected macrophages induces pyroptosis as well as promotes NLRP3 inflammasome-mediated processing of IL-1β (Kayagaki et al., 2011). Other studies have shown that AIM2 and NLRP3 inflammasomes can induce apoptosis through the activation of caspase-8 that interacts with the inflammasome complex via ASC (Sagulenko et al., 2013). Another recent study has shown that IL-1β is activated in a caspase-8 dependent manner in dendritic cells following fungal infection. Caspase-8 is activated in a non-canonical inflammasome complex that consists of CARD9, Malt1, Bcl-10, caspase-8 and ASC (Gringhuis et al., 2012). We summarized the two steps of canonical inflammasome activation (Fig. 1A) and also the non-canonical inflammasome activation (Fig. 1B).

Carcinomas (cancers derived from epithelial cells) and adenocarcinomas (cancers derived from epithelium in glandular tissues) are heterogeneous and consist of many cell types in addition to cancer cells, including cancer-associated fibroblasts, different tumor-infiltrating immune cells, adipocytes, endothelial cells, pericytes and others, all of which can secrete chemokines and cytokines (Grivennikov et al., 2010; Coussens et al., 2013). These chemokines and cytokines can directly affect cancer cells or cause cancer-associated inflammation by inducing immune cell infiltration. Myeloid cells, including immature myeloid cells, neutrophils and macrophages, represent one of the most frequent immune cells associated with cancer and a cellular source for inflammasome activation and secretion of IL-1β and IL-18. Cancer-associated inflammatory responses play roles in many aspects of cancer biology including tumor initiation, progression, metastasis, and treatment. In this review we will focus on the role of different inflammasomes in various malignancies and in tumor immune responses (Table 1).

**Table 1:** Studies on the roles of inflammasomes in inflammation and cancer

	Inflammasome components or mouse models	Impacts on inflammation and cancer	References
Gastric cancer	Stomach specific Transgenic expression of IL-1β	Induces stomach inflammation and tumorigenesis	Tu et al., 2008
	IL-1β	Promotes tumor growth and invasion	Li et al., 2012; Zhu et al., 2012
	*IL-18*^−/−^ mouse	Increased AOM/DSS-induced inflammation and tumorigenisis	Salcedo et al., 2010; Zaki et al., 2010b
	*Casp1*^−/−^ mouse	Increased AOM/DSS-induced inflammation and tumorigenisis	Allen et al., 2010; Zaki et al., 2010a; Dupaul-Chicoine et al., 2010
	*Asc*^−/−^ mouse	Increased AOM/DSS-induced inflammation and tumorigenisis	
Colon cancer	*Nlrp3*^−/−^ mouse	Increased AOM/DSS-induced inflammation and tumorigenisis	
	*Nlrc4*^−/−^ mouse	No discernible phenotype	
	*Nlrc4*^−/−^ mouse	Increased AOM/DSS-induced inflammation and tumorigenisis	Hu et al., 2011
	*Nlrp3*^−/−^ mouse	No discernible phenotype	
	*Nlrp6*^−/−^ mouse	Increased AOM/DSS inflammation and tumorigenesis	Chen et al., 2011; Elinav et al., 2011; Normand et al., 2011
	IL-1β	Promotes tumor growth, angiogenesis, invasion and metastasis	Dunn et al., 2012
	*Casp1*^−/−^ mouse	Reduced chemical induced tumorigenesis	Drexler et al., 2012
Melanoma	Myeloid cell-specific *Asc* deletion	Increased chemical-induced tumorigenesis	
	Keratinocyte-specific *Asc* deletion	Reduced chemical-induced tumorigenesis	
	NLRP3 inflammasome activation	Promotes progression and metastasis	Okamoto et al., 2010

## Gastrointestinal Cancers

Cancers of the gastrointestinal (GI) tract are frequently associated with chronic inflammation. The etiological link between tumorigenesis and the chronic inflammation is well documented for gastric cancer, which is associated with *Helicobacter pylori* infection and chronic gastritis, and colorectal cancer, which is frequently associated with inflammatory bowel disease (IBD) (Ferrone and Dranoff, 2010; Grivennikov et al., 2010; Zitvogel et al., 2012). As a mediator of inflammation, there is experimental evidence linking inflammasomes and their products to GI cancers.

IL-1β and IL-18, the major products of inflammasome activation, are pro-inflammatory cytokines and have been postulated to be pro-tumorigenic in inflammation-induced GI cancers. Chronic inflammation in the stomach due to *H. pylori* infection or other causes is mediated by the up-regulation of pro- and anti-inflammatory cytokines, including IL-1β and its naturally occurring receptor antagonist IL-1RN (Basso et al., 1996; McNamara and El-Omar, 2008). Other studies have shown that polymorphisms in the *IL-1β* and/or *IL-1RN* genes increase the risk of developing gastric cancer (El-Omar et al., 2000; Machado et al., 2001; Wang et al., 2007). Moreover, Tu et al. showed that stomach-specific expression of IL-1β in mice induces inflammation and tumorigenesis (Tu et al., 2008). While there is no direct evidence for the role of IL-1β in colitis-associated colon cancer (CAC), studies have indicated that IL-1β may promote tumor growth and invasion by inducing an epithelial to mesenchymal transition (EMT) and stem cell phenotype thus increasing the invasiveness of colon cancer cells (Li et al., 2012). Another study indicated that IL-1β stimulates COX-2 production in cancer associated fibroblasts, leading to an increase in the proliferation and invasive capabilities of colon cancer cells (Zhu et al., 2012). These studies suggest that IL-1β may promote colon tumor progression by acting on cells in the tumor microenvironment and/or cancer stem cells. IL-18, however, seems to play a tumor-suppressive role in CAC. IL-18 deficient mice have increased acute inflammation and tumorigenesis in an azoxymethane/dextran sodium sulphate (AOM/DSS) CAC model, a model that mimics the pathogenesis of IBD-driven colon tumorigenesis in human (Salcedo et al., 2010; Zaki et al., 2010b). In addition, recombinant IL-18 inhibited tumor progression in the AOM/DSS-induced CAC (Zaki et al., 2010b), suggesting a protective role for IL-18 from inflammation-promoted tumorigenesis in the colon. It has been suggested that IL-18 is critical for repairing colonic epithelium after injury, thus leading to an intact bacterial barrier and decreased inflammation (Zaki et al., 2010a).

There have been numerous studies on the role of individual inflammasome components in GI cancers; however, their specific roles may be context dependent. Caspase-1 expression is decreased in 19% of gastric cancer cases, which correlates with stage, lymph node metastasis and survival (Jee et al., 2005). In support of this etiological study, Allen et al. reported that *Casp1*^−/−^, *Asc*^−/−^, and *Nlrp3*^−/−^ mice had increased and recurring acute colitis and tumorigenesis relative to wild-type mice in the CAC model. In contrast, *Nlrc4*^−/−^ mice did not exhibit increased colitis and tumorigenesis (Allen et al., 2010). Furthermore, they reported that NLRP3-inflammasome activation in hematopoietic cells is critical for the tumor suppressive function (Allen et al., 2010), which has been attributed to IL-18 production (Salcedo et al., 2010; Zaki et al., 2010b). Interestingly, in contrast, Hu et al. reported that *Casp1*^−/−^ and *Nlrc4*^−/−^ mice, rather than *Nlrp3*^−/−^ mice, showed greater tumor load than wild-type mice (Hu et al., 2010, 2011). The tumor suppressor effect of the NLRC4-inflammasome seems to be primarily from NLRC4 expression in epithelial cells (Hu et al., 2011). Additionally, colonic epithelial cells deficient in caspase-1 bear greater resistance to apoptosis and increased proliferation relative to colon epithelial cells from wild-type mice (Hu et al., 2010). The tumor-suppressing function of inflammasomes does not always correlate with their role in promoting or suppressing DSS-induced inflammation; rather some studies have reported that *Casp1*^−/−^ and *Nlrp3*^−/−^ mice have decreased DSS-induced inflammation and colitis (Siegmund et al., 2001; Bauer et al., 2010).

NLRP6, another NLR family member, can also suppress tumorigenesis in the AOM/DSS CAC model, likely through secretion of IL-18 from hematopoietic cells (Chen et al., 2011), or regulation of wound healing by myofibroblasts (Normand et al., 2011). In support of this, a study by Ellinav et al. showed that *Nlrp6*^−/−^ mice had reduced IL-18 levels and exacerbated DSS-induced colitis compared to wild-type mice, and developed spontaneous colonic neoplasia. NLRP6, similar to NLRC4 (Hu et al., 2011), is also expressed in epithelial cells suggesting that NLRP6 may suppress tumorigenesis by the secretion of IL-18 from epithelial cells and not hematopoietic cells (Elinav et al., 2011). NLRP12 has also been implicated to play a role in inflammation and tumorigenesis in the colon. Studies have shown that *Nlrp12*^−/−^ mice are more susceptible to both DSS-induced colitis and AOM/DSS-induced tumorigenesis, indicating a tumor suppressor function for NLRP12 in CAC (Zaki et al., 2011; Allen et al., 2012). However, it is not known if NLRP6 or NLRP12 can form a functional inflammasome and further experimental support is required to make a definitive conclusion.

The inconsistencies between these various studies may be explained by differences in methodologies or animal facilities resulting in an altered composition of microbiota which can be sensed by different inflammasomes. The role of individual inflammasomes in inflammation-driven GI tumorigenesis may be specific to different inflammation-causing bacteria or their products present in the GI tract.

## Skin Cancers

The greatest environmental risk factor for skin cancer including melanoma is ultraviolet radiation which can promote cancer by causing DNA damage, immunosuppression and inflammation (Kanavy and Gerstenblith, 2011; Dunn et al., 2012). The involvement of inflammation in melanoma, the most malignant type of skin cancer, is indicated by the upregulation of inflammatory cytokines including IL-6, IL-8, CCL5, and IL-1β, all of which can be regulated by active IL-1β (Raman et al., 2007; Dinarello, 2009). Elevated level of active IL-1β in melanoma cells has been shown to act in both paracrine and autocrine manners to promote tumor growth, angiogenesis, recruitment of macrophages and immune suppresser cells, invasion and metastasis (Dunn et al., 2012). In addition, a recent study by Okamoto et al. indicates that the expression and secretion of active IL-1β in melanoma becomes increasingly autonomous during disease progression, which seems to be driven by constitutive activation of the NLRP3 inflammasome (Okamoto et al., 2010).

The function of active inflammasomes in skin cancer may differ depending on the cell type in which the inflammsomes are activated, as well as the stage of tumors. In a recent study, it was reported that while *Il1r1*^−/−^ and *Casp1*^−/−^ mice developed fewer tumors and had delayed tumor incidence compared to WT mice in a chemical-induced skin cancer model, *Asc*^−/−^ mice had no discernible phenotype when compared to WT animals. To further investigate the function of ASC in skin carcinogenesis, the investigators generated *Asc*-tissue-specific knockout mice in either keratinocytes or myeloid cells. The authors found that myeloid cell-derived ASC promotes tumor incidence while keratinocyte-derived ASC inhibits it. These results suggest a tissue specific role for inflammasomes in skin carcinogenesis, or ASC possesses other functions in keratinocytes to suppress tumorigenesis (Drexler et al., 2012). In a separate study, Liu et al. reported different functions for ASC in metastatic melanoma cells versus non-metastatic cells. In non-metastatic melanoma cells, ASC expression leads to decreased NF-κB activity and reduced tumorigenesis; however, ASC expression in metastatic melanoma cells increases NF-κB activity resulting in increased expression of pro-IL-1β, inflammasome mediated secretion of active IL-1β and enhances tumorigenesis (Liu et al., 2013). Previously it was reported that ASC can either inhibit or promote NF-κB activity depending on the expression of other PAAD/PYRIN-family proteins such as pyrin or NLRP3 (Stehlik et al., 2002). Therefore, it is possible that the effect of ASC expression in primary versus metastatic melanoma cells may be due to the differential expression of PAAD-family proteins.

## Inflammasome Activation In Other Cancers

In addition to GI and skin cancers, inflammasome activation may play important roles in other cancers including breast cancer and hepatitis C virus (HCV)-associated hepatocellular carcinoma (HCC). While there is no direct evidence showing the involvement of inflammasomes in breast cancer, it has been reported that IL-1β plays a role in tumorigenesis and progression of breast cancer. IL-1R1 expression is higher in ductal carcinoma *in situ* (DCIS) and invasive ductal carcinoma (IDC) relative to normal tissue (Pantschenko et al., 2003). Polymorphisms of IL-1R1 and IL-1β have been associated with disease progression and prognosis (Snoussi et al., 2005; Liu et al., 2010). Other studies have shown that elevated level of IL-1β in breast cancer is associated with a more aggressive phenotype and higher tumor grade (Jin et al., 1997; Chavey et al., 2007). In a mouse model of human breast cancer, fibroblast growth factor receptor 1-induced mammary tumorigenesis is associated with local production of IL-1β and can be blocked by treatment with an IL-1β neutralizing antibody (Reed et al., 2009).

The involvement of the inflammasome in HCC may depend on the carcinogen driving the process. Limited literature indicates that necrotic hepatocytes can secrete IL-1α, the major cytokine involved in diethylnitrosamine (DEN)-induced HCC (Sakurai et al., 2008). However, HCV infection, one of the most potent HCC inducers in human, activates the NLRP3 inflammasome in Kupffer cells (liver resident macrophages) and releases active IL-1β (Burdette et al., 2012; Negash et al., 2013). Although the HCV-induced HCC animal model is yet-to-be established, it is reasonable to speculate that HCV-associated IL-1β production may act in a similar manner as IL-1α for HCC development.

## Immune Regulation By Inflammasomes During Cancer Development and Therapy

Apart from distinct roles of inflammasomes in cancer mentioned above, inflammasome-mediated processing and secretion of IL-1β and IL-18 is critical in both innate and adaptive immune responses. Therefore, it is not surprising to find that inflammasomes can modulate tumor immunity during development and therapy (Eisenbarth and Flavell, 2009; Zitvogel et al., 2012). Studies have shown that IL-1β plays an important role in the expansion of myeloid derived suppressor cells (MDSC) in the bone marrow and their recruitment during chronic inflammation and tumorigenesis (Tu et al., 2008; Bunt et al., 2009; Elkabets et al., 2010; Sevko and Umansky, 2013). The role of inflammasomes in MDSC recruitment and immunosuppression is further supported by a study reporting that tumors derived from *Nlrp3*^−/−^ mice exhibit less infiltrating MDSC than those from WT mice (van Deventer et al., 2010). Tumor-associated MDSC may suppress natural killer (NK) cells, which would explain why loss of NLRP3 increases the anti-metastatic function of NK cells in a B16-F10 lung metastasis model (Chow et al., 2012). IL-18 can also suppress the anti-metastatic effect of NK-cells and IL-18 depletion boosts immune surveillance function of NK cells in B16-F10 melanomas and CT26 colon cancers (Terme et al., 2011). These studies indicate a role of inflammasomes in immunosuppression during tumorigenesis and metastasis.

Apart from their roles in immunosuppression during tumorigenesis and metastasis, inflammasomes may formulate the response to anti-tumor vaccines. A study by van Deventer et al. reported that *Nlrp3*^−/−^ mice had increased survival when treated with a dendritic cell (DC) derived vaccine against B16-F10 melanoma cells compared to their wild-type counterparts. This increase in survival of *Nlrp3*^−/−^ mice was associated with a decrease in the number of tumor-associated MDSC. Furthermore, depletion of MDSC in wild-type mice using an anti-GR-1 antibody improved survival in conjunction with the anti-tumor vaccine (van Deventer et al., 2010). This study suggests that the immunosuppressive role of the NLRP3 inflammasome may reduce the effectiveness of DC derived anti-tumor vaccines.

In addition to inducing apoptosis of cancer cells, some chemotherapeutic agents such as γ-irradiation, anthracyclines and oxaliplatin can induce pyroptosis which increases their efficiency at reducing tumor burden (Locher et al., 2010). Necrotic cell death results in the release of ATP from dying tumor cells into the extracellular space (Martins et al., 2009), which can be sensed by purinergic receptors on the surface of DCs, resulting in activation of tumor-antigen specific type-1 T helper cells (Th1) and cytotoxic CD8 T cells (Idzko et al., 2002; Elliott et al., 2009; Zitvogel et al., 2012). Activation of Th1 is mediated by ATP-dependent activation of NLRP3 inflammasome in DC and the release of IL-1β (Ghiringhelli et al., 2009; Iyer et al., 2009; Zitvogel et al., 2012). In line with the above evidence, anthracyclines and other chemotherapy agents cannot induce immunogenic cell death in mice lacking components of NLRP3 inflammasome including ASC, NLRP3, and Caspase-1 (Ghiringhelli et al., 2009). Furthermore, studies have shown that blocking IL-1β signaling reduces the anti-tumor effect of anthracyclines and oxaliplatin (Mattarollo et al., 2011).

## Perspective

The complex roles of inflammasome activation in cancer development or therapy have started to gain attention in recent years. During cancer development or treatment, inflammasomes can sense various signals including those not associated with cancer cells. Intestinal microbiota represents one of those non-cancer associated signals that may activate different inflammasomes with different potencies. It is thus not surprising that the same genetically manipulated animals in different animal facilities yield different, sometimes contrasting results in terms of inflammation and GI cancers. Composition of intestinal microbiota, however, may also affect cancers distal to the GI track due to the global effect of inflammasome activation and IL-1β or IL-18 secretion.

The roles of inflammasome activation in cancer are also tissue-specific. One good example is *Asc* depletion in a carcinogen-induced skin cancer model, where *Asc* depletion in myeloid cells inhibits skin tumor development but deletion in keratinocytes promotes it. In a colitis-associated colon cancer model, NLRP3 inflammasome works through myeloid cells whereas NLRC4 inflammasome plays a role in epithelial cells. Thus interpretation of the roles of different inflammasomes in cancer development has to take into account tissue types. Evaluation of the tissue specific roles of different inflammasome components will ultimately depend on the generation of conditional knockout mice for various molecules.

Compounds that target inflammasomes, such as IL-1β neutralizing antibodies, recombinant IL-1RN (anakinra) and IL-18 binding protein, have been developed, while others such as small molecule inhibitors that target caspase-1 are currently being developed (Green and Kroemer, 2005; Srivastava et al., 2010; Dinarello, 2011). The use of any therapy targeting the inflammasomes to treat cancer will depend on being able to determine when such therapies will be beneficial and when they will be detrimental due to the context specific function of the inflammasome in cancer. For example, blocking IL-1β signaling in metastatic melanoma cells and in breast cancer may be beneficial because IL-1β is indicated to promote tumor progression and metastasis in these tumors. However, blocking IL-1β may decrease the efficacy of the traditional chemotherapy agents if used in combination. Inflammasomes may provide a promising target for cancer therapy and prevention, though additional studies are required to determine the context-specific functions of inflammasomes during cancer development and progression.

## Abbreviations

CARD: caspase activation and recruitment domain
DAMPS: damage associated molecular patterns
IL: interleukin
NLR: Nod-like receptor
PAMPS: pathogen associated molecular patterns
ROS: reactive oxygen species
TLRs: toll-like receptors
TNF-α: tumor necrosis factor-α
